# Arthroscopic medial patellofemoral ligament reconstruction with polyethylene suture combined with medial retinaculum plication for the treatment of acute patellar dislocation in young and middle-aged patients with a follow-up of at least 2 years

**DOI:** 10.1186/s12891-024-07664-y

**Published:** 2024-07-25

**Authors:** Deding Liu, Dongdong Zhou, Zhengwei Zhu, Bao Zhang, Yongchao Zhang, Yaguang Zhao, Jiabing Lv, Jinzhong Zhao

**Affiliations:** 1 Department of Joint and Sports Injuries, Zhengzhou Central Hospital Affiliated Zhengzhou University, Zhengzhou, Henan 450000 China; 2 Department of Sports Medicine, Shanghai Sixth People’s Hospital, Shanghai Jiao Tong University, 600 Yishan Road, Shanghai, 200233 China

**Keywords:** Acute patellar dislocation, Medial patellofemoral ligament reconstruction, Medial retinaculum plication

## Abstract

**Purpose:**

The purpose of this study was to propose a surgical technique for arthroscopic medial patellofemoral ligament (MPFL) reconstruction with polyethylene suture combined with medial retinaculum plication and to evaluate the efficacy of this surgical technique in the treatment of acute patellar dislocation.

**Methods:**

Clinical data of patients with acute patellar dislocations treated with arthroscopic MPFL reconstruction with polyethylene tape (FiberTape) combined with medial support band compression were analyzed retrospectively from January 2018 to January 2021. The mean age of the patients was 25.15 ± 4.66 years; the mean follow-up time was 27.5 (24–36) months. Clinical evaluation consisted of apprehension test results, patellar extrapolation test results, Lysholm score, Kujala score, and IKDC score, the Patellar lateral shift distance and patellar tilt angle (PTA) measured by CT scan.

**Results:**

All patients had no recurrent patellar dislocation or subluxation after surgery, and the apprehension test was negative. In all patients, the Kujala score (36.0 ± 9.9 vs. 98.2 ± 3.1), the IKDC score (48.6 ± 7.0 vs. 90.6 ± 4.4) and the Lysholm score (32.8 ± 10.4 vs. 96.7 ± 3.1) had improved at the 24-month follow up (*P* < 0.05). In addition, PTA was significantly lower at the 12-month follow-up and 24-giving-month follow-up compared to the preoperative period (*P* < 0.05, Table 2). The patellar lateral shift distance decreased from 14.94 ± 6.11 mm preoperatively to 3.00 ± 1.40 mm (12-month follow up) and 3.26 ± 1.37 mm (24-month follow up), respectively.

**Conclusion:**

Arthroscopic MPFL reconstruction with polyethylene suture combined with medial retinaculum plication is a safe and reliable surgical technique for the treatment of acute patellar dislocation in young and middle-aged patients.

**Level of Evidence:**

Level III, Therapeutic Study.

## Introduction

Acute patellar dislocation is a common knee injury and the most common cause of traumatic hemarthrosis especially in children and adolescents [1–3]. Acute patellar dislocation can lead to injuries in various structures, including bones, cartilage, ligaments, and muscles [4, 5]. If not treated promptly and effectively, acute patellar dislocation can lead to knee dysfunction, such as recurrent dislocation, painful instability, anterior knee pain and patellofemoral degeneration [6, 7]. The traditional treatment protocol includes conservative treatment in the early stage, closed reduction, temporary fixation, and rehabilitation training, which is considered the standard treatment protocol [8]. However, for patients who do not undergo surgical treatment, the recurrence rate of acute patellar dislocation is high (30–70%), especially in young patients [9, 10]. A meta-analysis showed that surgical intervention can reduce the risk of recurrent dislocation for acute patellar dislocation patients [11]. A recent randomized controlled trial showed that for adolescent patients with first-time acute patellar dislocation and intra-articular loose bodies, medial patellofemoral ligament (MPFL) reconstruction surgery can significantly reduce the incidence of recurrent instability, reduce the need for subsequent surgery, and improve the patients’ athletic ability [12]. Moreover, some studies have shown that in cases of acute dislocation, MPFL is always ruptured or avulsed - mainly on the patellar side [13, 14]. Therefore, MPFL reconstruction surgery may be a good surgical option for adolescent patients with first-time acute patellar dislocation.

There are many grafts available for MPFL reconstruction, including autografts, allografts, and artificial ligaments [15]. Although MPFL reconstruction using these grafts can achieve good clinical results, complications such as graft relaxation, patellar track abnormality, recurrent dislocation, allograft tendon reaction, and donor pain can also be caused [16]. With the development of material research, synthetic materials have emerged as grafts for MPFL reconstruction. Previous studies have demonstrated that FiberTape (is an ultrahigh-strength tape, consisting of long-chain ultrahigh-molecular-weight polyethylene) is safe and effective, and provides higher initial strength than hamstring tendons [17, 18]. The main advantage of using synthetic materials for MPFL reconstruction is the preservation of the autologous tendon and therefore the reduction of donor complications [19].

Through surgical exploration of MPFL in patients with patellar dislocation, Tom et al. found that part of the MPFL was intact, but the length was longer than normal, and the original tension was lost [20], suggesting that early tightening of the medial retinaculum and restoring the tension of the medial retinaculum is also of great significance. Therefore, we aim to propose a surgical technique for MPFL reconstruction using polyethylene suture and medial retinaculum plication under arthroscopy for the treatment of acute patellar dislocation in young and middle-aged patients. We evaluated the clinical outcomes of this technique by comparing the preoperative and postoperative data and measuring the patellar position with magnetic resonance imaging (MRI) and computed tomography (CT) scans. We expect that this technique will enhance the knee stability after surgery and lead to good clinical results.

## Methods

### Patients

Clinical data of patients with acute patellar dislocations treated with arthroscopic MPFL reconstruction with polyethylene tape combined with medial support band compression were analyzed retrospectively from January 2018 to January 2021 at our hospital. The inclusion criteria were as follows: (1) patients with a first acute patellar dislocation between the ages of 18 and 40 years; (2) a mean time from injury to surgery of 3.18 ± 1.95 days; (3) a Caton-Deschamps index of 0.8–1.2 for the patella; and (4) a tibial tubercle-trochlear groove (TT-TG) distance of 10–20 mm. The exclusion criteria were as follows: (1) patients with recurrent patellar dislocations or severe trochlear dysplasia (Dejour types C and D); (2) male Q Angle > 15° or female Q Angle > 20°, femur forward at > 15°, knee valgus > 15°; (3) anterior and posterior cruciate ligament injuries or a history of knee surgery in the affected limb; (4) follow-up time less than 24 months; (5) Intra-articular cartilage fractures with fragments larger than 10 millimeters (> 10 mm) requiring surgical fixation; (6) Patellar chondral fractures requiring surgical repair and fixation. The mean age of the patients was 25.15 ± 4.66 years, and the mean follow-up time was 27.5 (24–36) months. These 33 patients included 18 males and 15 females, with 12 left knees and 21 right knees. The average operation time was 56.91 ± 10.86 min. This research has been approved by the institutional review board (IRB) of the authors’ affiliated institutions.

### Surgical technique

All patients were placed in the supine position under general anesthesia, and no tourniquet was used during the operation. A diagnostic arthroscopy was performed using standard anteromedial, anterolateral, and superolateral patellar portals.

**Fig. 1:**
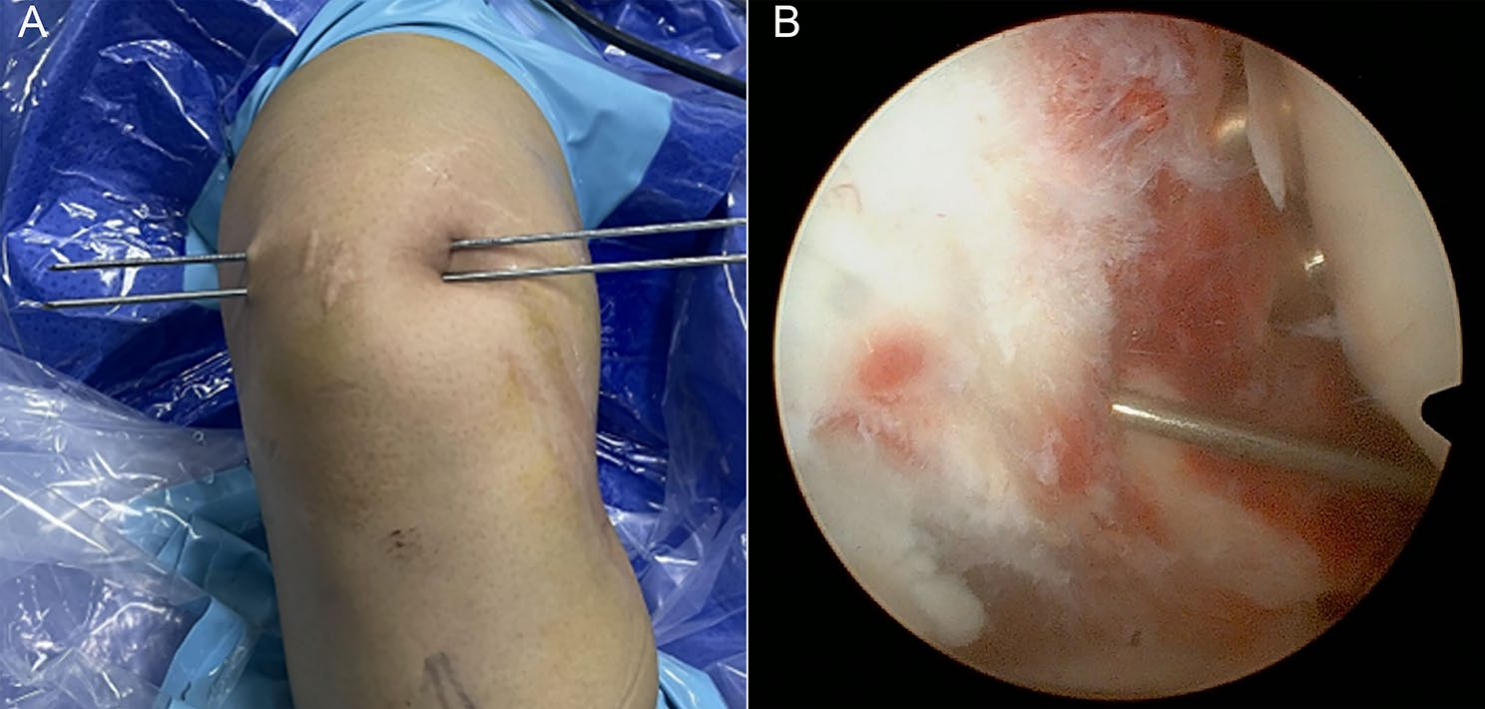
(**A**) In vitro 2.4 mm Kirschner wire with a 5 mm interval. (**B**) The exchange rod opens the torn medial patellar supporting band, revealing the placement of two 2.4 mm Kirschner wires

Intraoperatively, loose bodies within the joint were removed under arthroscopic visualization, and the patellar tracking was assessed. Externally, the upper and lower poles of the patella and the widest part of the patella (usually located at the junction of the upper one-third of the patella and the attachment site of the MPFL) were palpated. With the knee flexed at 30°, a 5 mm incision was made at the widest part of the medial edge of the patella. Two 2.5 mm tunnels were drilled vertically from the intersection of the medial border of the patella and the articular surface towards the midline of the patella, with a 5 mm distance between the tunnels, using a 2.5 mm Kirschner wire through this incision (Fig. 1). A 2.4 mm trailing hole guide wire was used to introduce a polydioxanone (PDS) sutures (W9236, Ethicon) into the tunnels.

**Fig. 2:**
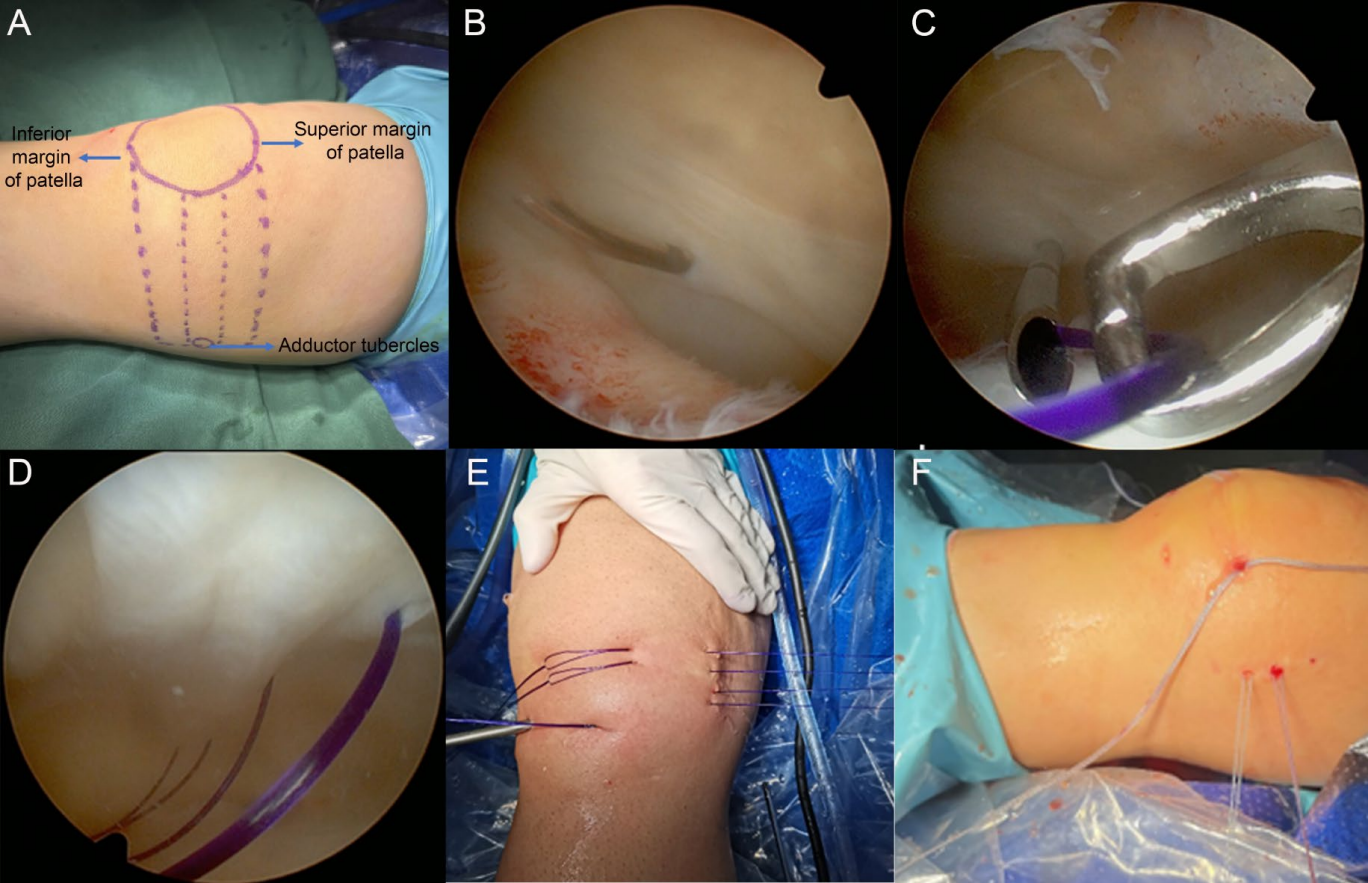
(**A**) Lateral schematic view of the quadrilateral region. (**B**) The epidural puncture needle passes from the outer to the inner edge of the medial supporting band. (**C**) The PDS traction wire is inserted through the epidural puncture needle. (**D**) The arthroscope is positioned in the lateral view, showing the upper edge of the patella, the patellar apex, and four PDS traction wires placed horizontally along the tripartite line. (**E**) Distribution of the external PDS traction wires. (**F**) The FiberTape forms a U-shaped loop around the surface of the patella. Knots are tied between sutures 1 and 3, and between sutures 2 and 4 in the superficial fascial layer. The suture ends are cut, and the alternating traction of the free ends of the sutures on the lateral side of the patella tightens the suture knots, completing the tightening of the medial supporting band

**Fig. 3:**
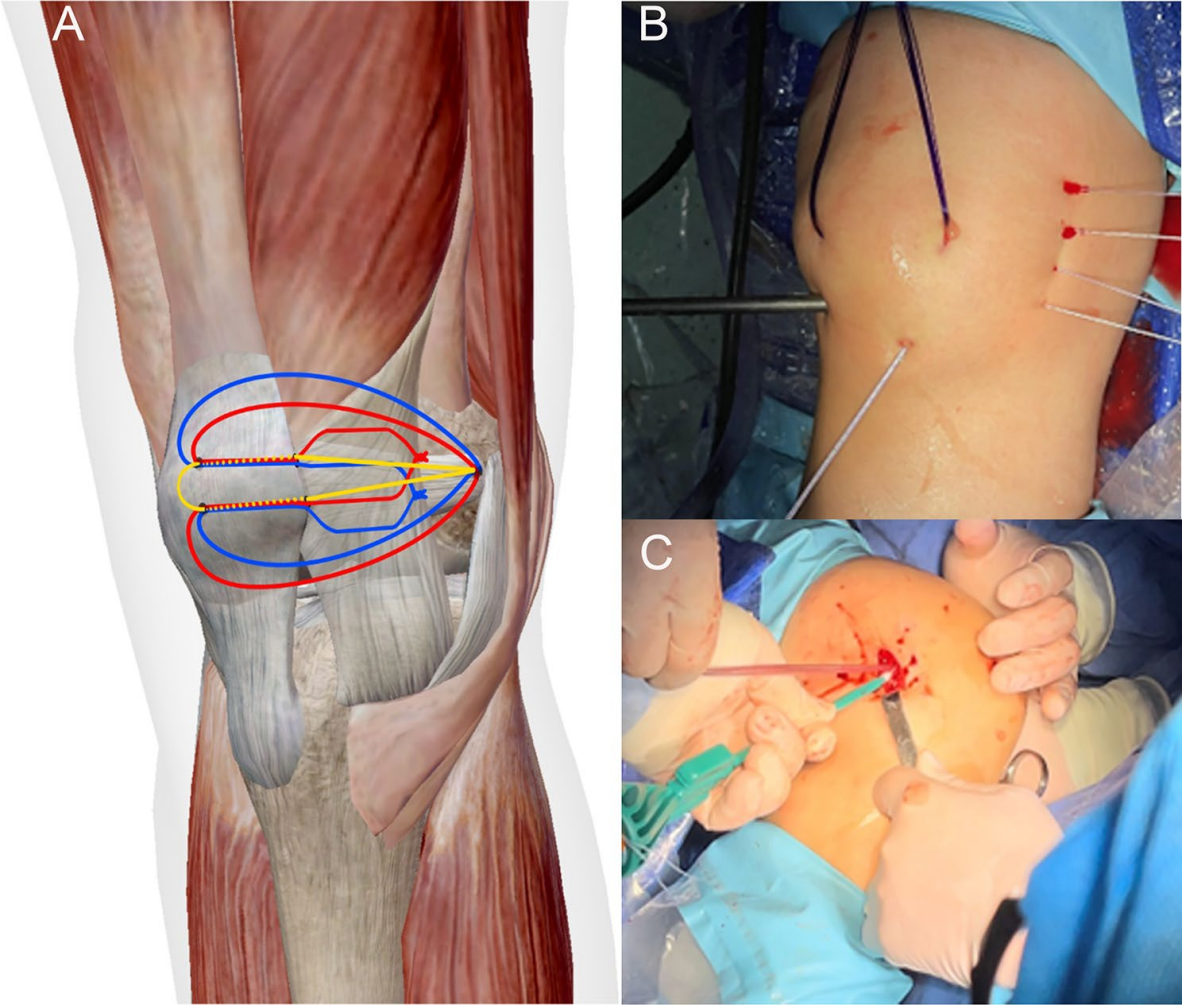
(**A**) The PDS traction wires pull four high-strength sutures into the joint to tighten the medial supporting band. (**B**) The knot tying of sutures at suture 1 and 3, and suture 2 and 4, completes the tightening of the medial support band. The FiberTape is passed through the near and far tunnels of the patella, then folded back on the patellar surface, and collectively secured at the MPFL insertion point. The red color represents Johnson & Johnson high-strength sutures, named suture 1 and 3; the blue color represents Ethicon high-strength sutures, named suture 2 and 4, while the yellow color represents FiberTape. (**C**) Positioned in the depression between the medial femoral condyle and the adductor tubercle, a bone tunnel is prepared obliquely from the proximal, anterior, and lateral sides using an outside-in drilling tool, followed by tightening and fixation of the six suture ends using outside-in screws at 30° of knee flexion

Medial retinaculum plication: with the patient’s knee extended, a quadrilateral shape was marked with the medial border of the patella as the lateral side, the longitudinal axis of the adductor tubercle as the medial side, the upper border of the patella as the superior boundary, and the horizontal line at the level of the inferior pole of the patella as the inferior boundary. The quadrilateral was divided into three equal parts horizontally (Fig. 2A). PDS sutures were introduced through the four horizontal lines of the quadrilateral using a dural puncture needle (Fig. 2B), along with two No. 2 high-strength sutures (Orthocord; DePuy Mitek, Raynham, MA) (named as suture 1 and suture 3) and two No. 2 high strength sutures (UltraBraid; Smith & Nephew Endoscopy, Andover, MA) (named as suture 2 and suture 4), which were passed from the outside of the joint to the inside through the PDS sutures (Fig. 2C) and then pulled out through the incision on the medial border of the patella (Fig. 2D). One end of suture 1 and suture 2, as well as one end of a 2-mm FiberTape (AR-7237-7, Arthrex FiberTape, Arthrex), were pulled out through the proximal patellar tunnel (Fig. 2B), while the other end of suture 3 and suture 4, along with the other end of the FiberTape, were pulled out through the distal patellar tunnel (Fig. 2C, D and E). The FiberTape formed a U-shaped loop around the patellar surface. Two 2 mm incisions were made at the locations of suture 2 and suture 3 on the medial side, and complete subfascial dissection was performed in the area of the MPFL. The ends of each suture were pulled out through the subfascial layer through these incisions, and suture suture 1 and suture 3, as well as suture 2 and suture 4, were tightened and tied in the subfascial layer (Fig. 2F). The suture tails were folded back along the patellar surface and pulled out through the incision on the medial edge of the patella. The tails of sutures 1–4 were then tensioned to complete the tightening of the medial support band (Fig. 3A).

MPFL reconstruction: One end of a FiberTape was pulled out through the proximal patellar tunnel, and the other end was pulled out through the distal patellar tunnel. The FiberTape formed a U-shaped loop around the patellar surface. Sutures 1–4 are also pulled into the patellar bony channel after being knotted and later secured to the femoral stop of the MPFL (Fig. 3B), which serves the function of tightening the medial support band while also taking care of the function of the MPFL.

The ends of sutures 1 to 4 were folded back along the surface of the patella and brought out through the incision on the medial border of the patella, along with the FiberTape that encircled the patella in a U-shaped manner. With the knee flexed at 90°, a longitudinal incision of approximately 1.5 cm was made on the surface that between the medial epicondyle and the adductor tubercle, until the adductor tubercle, the medial epicondyle of the femur, and the gastrocnemius tubercle were exposed. The ends of sutures 1 to 4 and the FiberTape were pulled out through the superficial and deep fascia through this incision. The six sutures were tightened with the knee fully extended, and then the knee was gradually flexed to 120° to assess the degree of medial patellar translation, tension of the lateral support band, and patellar reduction. If the medial patellar translation was too small or the reduction was unsatisfactory, or if the tension of the lateral tissues was too high, the lateral support band was released. However, lateral support band release was prohibited for patients with excessive or normal medial patellar translation. Finally, using a drill guide, a bone marrow canal was prepared obliquely towards the anterolateral, anterior, and lateral sides between the medial epicondyle of the femur and the adductor tubercle. The six sutures were tightened and secured with an interference screw at 30° of knee flexion (Fig. 3C).

### Postoperative rehabilitation

After the operation, the affected limb was routinely treated with ice compress and pressure dressing, the knee joint adjustable brace was used for fixation, and non-steroidal anti-inflammatory drugs were taken orally. Isometric contraction exercise of the quadriceps femoris was performed after awakening from anesthesia. The patella was pushed inward at regular intervals to prevent adhesion. Passive flexion exercise was performed on the first day after the operation. After 3 weeks, active knee flexion exercise was started, and the knee flexion Angle was limited to 0–90° within 3 weeks. After 6 weeks, a full range of active and passive activities could be performed, and the brace was removed. They could return to normal life after 3 months. Physical activity was gradually initiated after 6 months.

### Outcome measures

The apprehension test results, lateral patellar translation grade (translation to 1/4, 1/2, 3/4, and 1 of the patellar width was documented as grade I, II, III, and IV, respectively; lateral translation greater than the whole patellar width was documented as grade V; lateral translation grade greater than III was considered as abnormal) [21], Lysholm score [22], Kujala score [23], and International Knee Documentation Committee (IKDC) score [24] was recorded before the operation, at 12 months and 24 months after the operation to evaluate the clinical results. In terms of imaging, the Patellar Tilt Angle (PTA), Congruence Angle (CA), and lateral patellar translation were assessed using CT scans at 30 degrees of knee flexion preoperatively, as well as at 12 and 24 months postoperatively, following the methodology described in the previous study [21]. The lateral shift distance of the patella was defined as the distance from the intersection of the transverse axis of the patella and the lateral line of the femoral condyle to the lateral edge of the patella. The transverse axis of the patella, the lateral line of the femoral condyle, and the lateral edge of the patella were determined by the image of the largest width of the patella [28]. The patients were graded as excellent, very good, good, and poor according to the Crosby-Insall scale (pain, joint function and return to sport). Moreover, knee MRI was also performed preoperatively and postoperatively in all patients presenting with acute patellar dislocation, according to the previous method [25].

### Statistical analysis

SPSS 21.0 software was used for statistical analysis, and the measurement data were expressed as mean ± sd. One-way analysis of variance was used when the measurement data were normally distributed, and the rank sum test was used when the data were not normally distributed. The *Wilcoxon* rank sum test was used for ranked data. *P* < A difference of 0.05 was considered statistically significant.

## Results

During the study period, 33 patients (33 knees) met the inclusion criteria and were included in this study (Table 1). The mean intraoperative blood loss was 22.56 ± 5.02 ml. The average length of hospital stay was 4.36 ± 1.39 days. The grade of trochlear dysplasia was classified using Dejour classification. The trochlea form was classified as Dejour-A in 32 cases and Dejour-B in 1 case. Patellar morphology was evaluated according to Wiberg classification. There were one Wiberg type I patella, 28 type II, and 4 type III.

**Table 1:** Characteristics of the patients

Variable	All patients, *n* = 33
Age, years, mean ± SD	25.15 ± 4.66
Females/males	15/18
Right/left knees	21/12
Operation time	56.91 ± 10.86
Blood loss	22.56 ± 5.02
Hospital stay	4.36 ± 1.39
Follow-up time, month, mean ± SD	27.48 ± 3.28
Sports activity	
Military training	12
Football	7
Basketball	11
Falling	3

All patients had a positive preoperative apprehension test, and the lateral patellar translation grade results showed 23 patients (69.7%) with grade II and 10 patients (30.3%) with grade III. At 12 months after surgery, the lateral patellar translation grade results showed that 22 patients (66.7%) were grade I and 11 patients (33.3%) did not have lateral patellar translation, which was significantly improved compared with that before operation (*P* < 0.05). At 24 months after surgery, lateral patellar translation grade showed that 26 patients (78.8%) were grade I and 7 patients (21.2%) did not have lateral patellar translation, which was significantly improved compared with that before operation (*P* < 0.05). All patients had no recurrent patellar dislocation or subluxation after surgery, and the apprehension test was negative. In all patients, the Kujala score (36.0 ± 9.9 vs. 98.2 ± 3.1), the IKDC score (48.6 ± 7.0 vs. 90.6 ± 4.4) and the Lysholm score (32.8 ± 10.4 vs. 96.7 ± 3.1) had improved at the 24-month follow up (*P* < 0.05, Table 2).

**Table 2:** Preoperative, 12-month follow-up and 24-month follow-up functional comparisons

Variables	Preoperative	12-month follow-up	24-month follow-up	*P* value
Lysholm score	32.8 ± 10.4	89.3 ± 4.8	96.7 ± 3.1	< 0.01
Kujala score	36.0 ± 9.9	95.4 ± 7.6	98.2 ± 3.1	< 0.01
IKDC score	48.6 ± 7.0	88.4 ± 4.5	90.6 ± 4.4	< 0.01
Patellar lateral shift distance	14.94 ± 6.11	3.00 ± 1.40	3.26 ± 1.37	< 0.01
Patellar tilt angle	28.4 ± 4.5	16.7 ± 3.0	16.5 ± 2.8	< 0.01

Data are expressed as mean values ± standard deviation

At the 12-month follow up, 1 patient had occasional morning pain, 5 patients had mild pain after extensive activity, all patients had no limitation of knee flexion function, and 27 patients returned to pre-injury sports level. At the 12-month follow-up, 27 (81.8%) patients rated as “excellent”, 3 (9.1%) as “very good”, and 3 (9.1%) as “good”. At the 24-month follow-up, 3 patients had mild pain after strenuous activity, and all patients were able to return to preoperative normal exercise levels. At the 24-month follow-up, 31 patients (93.9%) were rated as “excellent” and 2 patients (6.1%) were rated as “very good”.

**Fig. 4:**
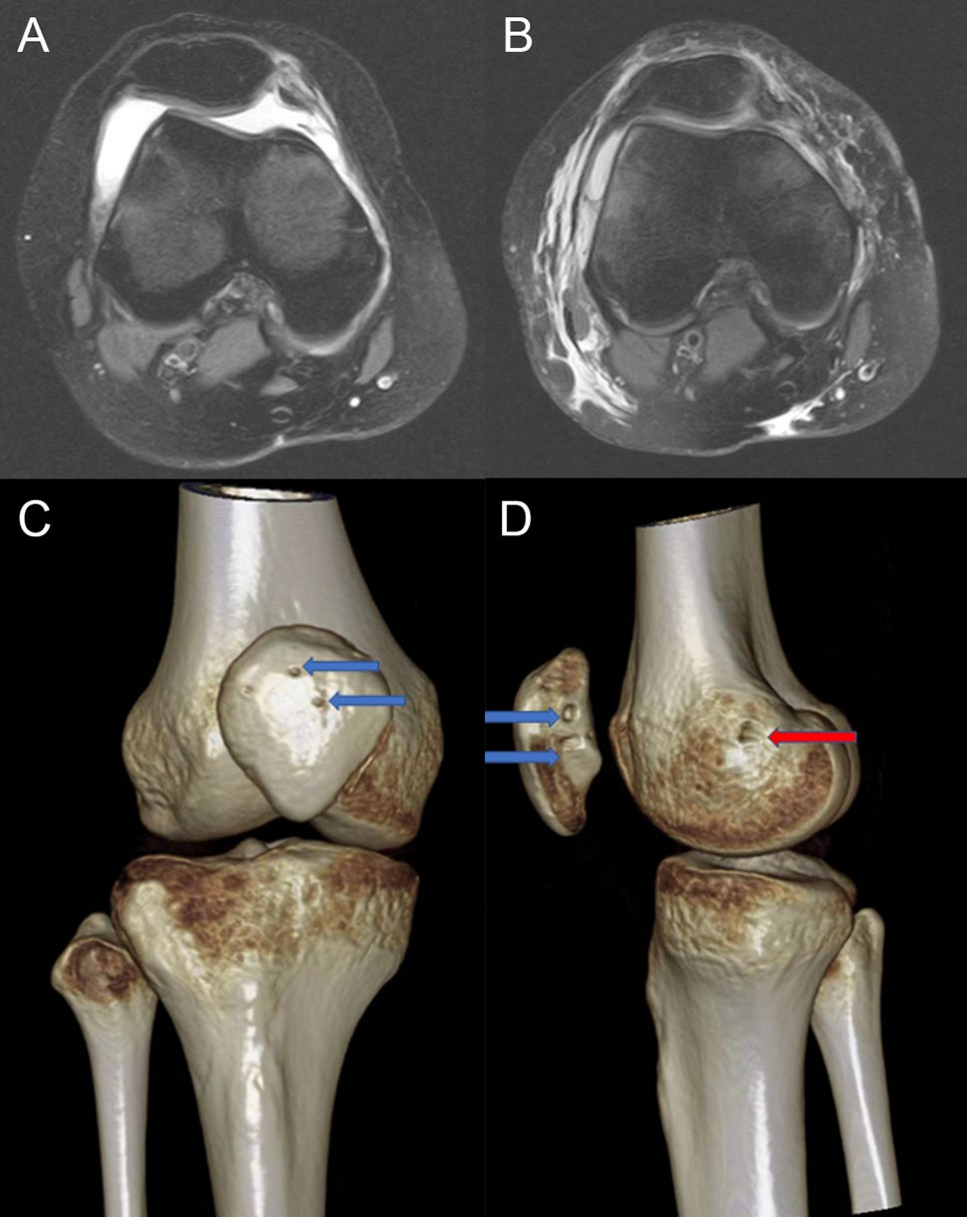
(**A**) Preoperative MRI showed lateral dislocation of the patella. (**B**) Postoperative MRI showed that the continuity of the MPFL was restored. Postoperative CT showed the location of the patellar canal (blue arrow) (**C**) and the external row nail (red arrow) (**D**)

Preoperative MRI showed loss of continuity of the MPFL and lateral dislocation of patella, and postoperative MRI showed that MPFL continuity restoration and good patellofemoral joint alignment (Fig. 4). PTA was significantly lower at the 12-month follow-up and 24-giving-month follow-up compared to the preoperative period (*P* < 0.05, Table 2). The lateral patellar translation decreased from 14.94 ± 6.11 mm preoperatively to 3.00 ± 1.40 mm (12-month follow up) and 3.26 ± 1.37 mm (24-month follow up), respectively.

## Discussion

The recurrence of patellar dislocation after treatment has a certain relationship with the initial treatment methods. Early and targeted treatment can reduce the probability of recurrence [26]. MPFL is the most important anatomical structure to maintain the position of the patella and limit its lateral dislocation [27, 28]. Therefore, restoring the limiting force on the medial side of the patella is considered crucial for any kind of stabilization of the patella and prevention of recurrence of the dislocation. This study is the first time to propose arthroscopic MPFL reconstruction with polyethylene suture combined with medial retinaculum plication for the treatment of acute primary patellar dislocation. Following a minimum of two years of clinical follow-up, the efficacy of this technique has been substantiated.

At present, the treatment of acute patellar dislocation is still controversial. Some researchers believe that acute patellar dislocation should be treated conservatively [29]. For acute patellar dislocation, conservative treatment is generally based on physical therapy, including plaster fixation, bracing fixation, and functional exercise [30]. One study found that only 26.4% of patients were able to participate in sports activities without restriction after conservative treatment [31]. In addition, the recurrence rate of dislocation after conservative treatment of patients has been reported to be as high as 50% [32]. Therefore, surgical treatment is recommended for young, active patients with heavy physical labor and sports, as well as for patients with osteochondral fractures that need reduction, free osteochondral fragments that need to be removed, and patients with severe damage to the medial stable structure.

MPFL reconstruction is considered to be the most important soft tissue structure for limiting patella dislocation and maintaining patella stability [33]. Therefore, MPFL reconstruction is one of the routine procedures for the treatment of recurrent patella dislocation. Some researchers believe that due to the diversity of MPFL injury forms, MPFL reconstruction alone cannot reduce the incidence of patellar re-dislocation [34]. Therefore, in this study, the surgical technique of MPFL reconstruction combined with medial retinaculum plication was used to treat acute primary patellar dislocation. Medial retinaculum plication is a traditional surgical procedure for the treatment of acute patellar dislocation, which is widely used especially in children and adolescents. However, there are still controversies in surgical indications, surgical techniques and re-dislocation rate [35]. Hautamaa has shown in fresh cadavers that tight suture of the medial retinacula plays an important role in correcting the lateral dislocation of the patella [36]. Nha et al. used the medial retinaculum plication under arthroscopy and determined the tension of the medial retinaculum according to the patellar track after reduction with satisfactory clinical results [37]. Patients with an initial acute patellar dislocation often present with anatomical abnormalities, and conservative treatment is associated with a high recurrence rate [38]. In our study, we propose that for young individuals experiencing their first acute patellar dislocation, due to their inherent capacity for self-healing, a combined medial support band tightening should be implemented within the quadrilateral area formed by the medial edge of the patella, the adductor tubercle, and the upper and lower edges of the patella. This area encompasses critical medial stabilizing structures such as the fibers of the vastus medialis oblique muscle, the MPFL, and the medial patellotibial ligament. Tightening and repairing these structures at the time of initial dislocation can effectively preserve the body’s original stabilizing structures. During the operation, the 1#-4# high-strength suture was used to cross knot, which could avoid the suture cutting on the medial retinaculum and lead to contractive failure. At the same time, the suture was pulled out through the patellar tunnel and fixed on the femoral insertion together with the polyethylene suture band reconstructed by MPFL. In a certain sense, the repair and enhancement of the patellar insertion, body and femoral insertion of the medial retinaculum were taken into care. It can form a complementary enhancement with MPFL reconstruction. For young and middle-aged patients experiencing their first acute patellar dislocation, this technique promotes the repair of the medial stable structure while minimizing interference with adjacent normal structures. The combined operation proposed in this study may be one of the ways to solve this problem. The kujala score, IKDC score and Lysholm score were significantly improved, and the patellar tilt Angle was significantly reduced. At 24 months after operation, all patients could reach the preoperative normal movement level.

The source of materials needed for MPFL reconstruction is a problem that is considered by doctors and patients. The grafts for MPFL reconstruction mainly include autologous tendons, allogeneic tendons, and artificial ligaments [39]. Allogeneic tendons have problems such as rejection and high price [40], artificial ligaments have complications such as synovitis and ligament relaxation [41], and autologous tendons have complications such as pain in the tendon extraction area, hematoma formation, and neurovascular injury [42]. A polyethylene suture band may be a valuable option to restore patellar stability without the need to harvest autogenous tendon grafts. Polyethylene suture bands can protect the repaired native ligament during its biological healing and prevent increased patellar lateralization [43]. Tsushima T et al. compared the mechanical strength of polyethylene strip and semitendinosus tendon in MPFL reconstruction from the perspective of biomechanics, and the test showed that the strength of polyethylene strip was better than that of autologous tendon [17]. Lee PYF et al. found that there was no significant difference in the efficacy of MPFL reconstruction between polyethylene suture and autologous vastus muscle [18], but polyethylene suture has small volume, light foreign body sensation, good histocompatibility, and high patient satisfaction, which was consistent with our results of MPFL reconstruction with polyethylene suture.

There are some limitations in this study. Firstly, this study is a retrospective analysis, the sample size is small, and there is no comparison of the efficacy of the new operation with MPFL reconstruction and medial retinaculum plication. Secondly, the patients were followed up for a short time. Although the mid-term effect was satisfactory, the long-term effect could not be evaluated, and further follow-up was needed. Thirdly, the study did not incorporate a control group. Finally, the MPFL reconstruction materials used in this study are not widely used at home and abroad, and lack of long-term follow-up results of large cases.

## Conclusion

The arthroscopic MPFL reconstruction with polyethylene suture combined with medial retinaculum plication is a safe and reliable techniques for the treatment of acute patellar dislocation. However, the number of cases in this study is small, and large samples and long follow-up are needed to verify.

## Data Availability

The data used and analyzed in this study are available upon request from the corresponding author.

## References

[1] MacDonald J, Rodenberg R, Sweeney E. Acute knee injuries in children and adolescents: a review. JAMA Pediatr. 2021;175(6):624–30. 33749718 10.1001/jamapediatrics.2020.6130

[2] Atkin DM, Fithian DC, Marangi KS, Stone ML, Dobson BE, Mendelsohn C. Characteristics of patients with primary acute lateral patellar dislocation and their recovery within the first 6 months of injury. Am J Sports Med. 2000;28(4):472–9. 10921637 10.1177/03635465000280040601

[3] Vetrano M, Oliva F, Bisicchia S, Bossa M, De Carli A, Di Lorenzo L, Erroi D, Forte A, Foti C, Frizziero A, et al. I.S.Mu.L.T. First-time patellar dislocation guidelines. Muscles Ligaments Tendons J. 2017;7(1):1–10. 28717605 10.11138/mltj/2017.7.1.001 PMC5505576

[4] Panni AS, Cerciello S, Maffulli N, Di Cesare M, Servien E, Neyret P. Patellar shape can be a predisposing factor in patellar instability. Knee Surg Sports Traumatol Arthrosc. 2011;19(4):663–70. 21153544 10.1007/s00167-010-1329-4

[5] Migliorini F, Eschweiler J, Betsch M, Knobe M, Tingart M, Maffulli N. Prognostic factors for isolated medial patellofemoral ligament reconstruction: a systematic review. Surgeon. 2022;20(4):e112–21. 33962891 10.1016/j.surge.2021.03.003

[6] Matic GT, Magnussen RA, Kolovich GP, Flanigan DC. Return to activity after medial patellofemoral ligament repair or reconstruction. Arthroscopy. 2014;30(8):1018–25. 24768468 10.1016/j.arthro.2014.02.044

[7] Sanders TL, Pareek A, Hewett TE, Stuart MJ, Dahm DL, Krych AJ. High rate of recurrent patellar dislocation in skeletally immature patients: a long-term population-based study. Knee Surg Sports Traumatol Arthrosc. 2018;26(4):1037–43. 28299386 10.1007/s00167-017-4505-y

[8] Nwachukwu BU, So C, Schairer WW, Green DW, Dodwell ER. Surgical versus conservative management of acute patellar dislocation in children and adolescents: a systematic review. Knee Surg Sports Traumatol Arthrosc. 2016;24(3):760–7. 26704809 10.1007/s00167-015-3948-2

[9] Palmu S, Kallio PE, Donell ST, Helenius I, Nietosvaara Y. Acute patellar dislocation in children and adolescents: a randomized clinical trial. J Bone Joint Surg Am. 2008;90(3):463–70. 18310694 10.2106/JBJS.G.00072

[10] Fithian DC, Paxton EW, Stone ML, Silva P, Davis DK, Elias DA, White LM. Epidemiology and natural history of acute patellar dislocation. Am J Sports Med. 2004;32(5):1114–21. 15262631 10.1177/0363546503260788

[11] Erickson BJ, Mascarenhas R, Sayegh ET, Saltzman B, Verma NN, Bush-Joseph CA, Cole BJ, Bach BR Jr. Does Operative Treatment of First-Time patellar dislocations lead to increased Patellofemoral Stability? A systematic review of overlapping Meta-analyses. Arthroscopy. 2015;31(6):1207–15. 25636989 10.1016/j.arthro.2014.11.040

[12] Gurusamy P, Pedowitz JM, Carroll AN, Johnson K, Chambers HG, Edmonds EW, Pennock AT. Medial Patellofemoral Ligament Reconstruction for adolescents with Acute First-Time patellar dislocation with an Associated Loose body. Am J Sports Med. 2021;49(8):2159–64. 34097530 10.1177/03635465211013543

[13] Duthon V. Acute traumatic patellar dislocation. Orthop Traumatology: Surg Res. 2015;101(1):S59–67. 10.1016/j.otsr.2014.12.001 25592052

[14] Abdel-Aziz A, Sherif MM, Waly MR, Abdel-Aziz MA, Mostafa Zaky Abdelrazek BH. Simple cost-effective reinsertion of Avulsed Medial Patellofemoral Ligament in Acute Patellar dislocation. Arthrosc Tech. 2021;10(3):e847–53. 33738223 10.1016/j.eats.2020.10.076 PMC7953263

[15] Witoński D, Kęska R, Synder M, Sibiński M. An isolated medial patellofemoral ligament reconstruction with patellar tendon autograft. *BioMed Research International* 2013, 2013. 10.1155/2013/637678 PMC3810442 24224173

[16] Shah JN, Howard JS, Flanigan DC, Brophy RH, Carey JL, Lattermann C. A systematic review of complications and failures associated with medial patellofemoral ligament reconstruction for recurrent patellar dislocation. Am J Sports Med. 2012;40(8):1916–23. 22679297 10.1177/0363546512442330 PMC3615712

[17] Tsushima T, Tsukada H, Sasaki S, Naraoka T, Yamamoto Y, Tsuda E, Ishibashi Y. Biomechanical analysis of medial patellofemoral ligament reconstruction: FiberTape^®^ with knotless anchors versus a semitendinosus tendon autograft with soft anchors. J Orthop Sci. 2019;24(4):663–7. 30573394 10.1016/j.jos.2018.11.018

[18] Lee PYF, Golding D, Rozewicz S, Chandratreya A. Modern synthetic material is a safe and effective alternative for medial patellofemoral ligament reconstruction. Knee Surg Sports Traumatol Arthrosc. 2018;26(9):2716–21. 28929187 10.1007/s00167-017-4711-7

[19] Xu JC, Zhang BX, Jia YF, Wang XF, Shen K, Ren WB, Sun R. Medial Patellofemoral Ligament Reconstruction using suture tape for Patellofemoral Joint instability. Orthop Surg. 2021;13(3):847–54. 33749146 10.1111/os.12945 PMC8126912

[20] Tom A, Fulkerson JP. Restoration of native medial patellofemoral ligament support after patella dislocation. Sports Med Arthrosc Rev. 2007;15(2):68–71. 17505320 10.1097/JSA.0b013e31803035d3

[21] Zhao J, Huangfu X, He Y, Liu W. Recurrent patellar dislocation in adolescents: medial retinaculum plication versus vastus medialis plasty. Am J Sports Med. 2012;40(1):123–32. 21900625 10.1177/0363546511420551

[22] Kocher MS, Steadman JR, Briggs KK, Sterett WI, Hawkins RJ. Reliability, validity, and responsiveness of the Lysholm knee scale for various chondral disorders of the knee. J Bone Joint Surg Am. 2004;86(6):1139–45. 15173285 10.2106/00004623-200406000-00004

[23] Kujala UM, Jaakkola LH, Koskinen SK, Taimela S, Hurme M, Nelimarkka O. Scoring of patellofemoral disorders. Arthroscopy. 1993;9(2):159–63. 8461073 10.1016/S0749-8063(05)80366-4

[24] Hefti F, Müller W, Jakob RP, Stäubli HU. Evaluation of knee ligament injuries with the IKDC form. Knee Surg Sports Traumatol Arthrosc. 1993;1(3–4):226–34. 8536037 10.1007/BF01560215

[25] Friedman MV, Hillen TJ, Misra S, Hildebolt CF, Rubin DA. Quantitative variable assessment of patellar instability: an MRI-based study. Am J Roentgenol. 2020;215(5):1163–70. 32901564 10.2214/AJR.19.22556

[26] Wen Tian F, Wang J, Huang L, Chen, Shiqiang Hu, Peng A. Meta analysis on the treatment of acute patellar dislocation by surgical versus conservative treatment. Chin J Tissue Eng Res. 2012;16(44):8228–34.

[27] Wang C-h, Ma L-f, Zhou J-w, Ji G, Wang H-y, Wang F, Wang J. Double-bundle anatomical versus single-bundle isometric medial patellofemoral ligament reconstruction for patellar dislocation. Int Orthop. 2013;37(4):617–24. 23371425 10.1007/s00264-013-1788-6 PMC3609965

[28] Migliorini F, Marsilio E, Oliva F, Eschweiler J, Hildebrand F, Maffulli N. Chondral injuries in patients with recurrent patellar dislocation: a systematic review. J Orthop Surg Res. 2022;17(1):63. 35101078 10.1186/s13018-022-02911-1 PMC8802427

[29] Apostolovic M, Vukomanovic B, Slavkovic N, Vuckovic V, Vukcevic M, Djuricic G, Kocev N. Acute patellar dislocation in adolescents: operative versus nonoperative treatment. Int Orthop. 2011;35(10):1483–7. 21574051 10.1007/s00264-011-1265-z PMC3174298

[30] Petri M, Ettinger M, Stuebig T, Brand S, Krettek C, Jagodzinski M, Omar M. Current concepts for patellar dislocation. Arch Trauma Res. 2015;4(3):e29301. 26566512 10.5812/atr.29301 PMC4636822

[31] Ihle C, Maurer J, Ziegler P, Stöckle U, Ateschrang A, Ahrend M-D, Schröter S. Sporting activity is reduced following medial reefing performed for patellar dislocation. BMC Musculoskelet Disord. 2019;20(1):1–10. 30669997 10.1186/s12891-019-2400-z PMC6343311

[32] Krebs C, Tranovich M, Andrews K, Ebraheim N. The medial patellofemoral ligament: review of the literature. J Orthop. 2018;15(2):596–9. 29881201 10.1016/j.jor.2018.05.004 PMC5990246

[33] Migliorini F, Pilone M, Eschweiler J, Marsilio E, Hildebrand F, Maffulli N. High rates of damage to the Medial Patellofemoral ligament, lateral trochlea, and Patellar Crest after Acute Patellar dislocation: magnetic resonance imaging analysis. Arthroscopy. 2022;38(8):2472–9. 35157964 10.1016/j.arthro.2022.01.044

[34] Mariani PP, Liguori L, Cerullo G, Iannella G, Floris L. Arthroscopic patellar reinsertion of the MPFL in acute patellar dislocations. Knee Surg Sports Traumatol Arthrosc. 2011;19(4):628–33. 21063679 10.1007/s00167-010-1315-x

[35] Cerciello S, Lustig S, Costanzo G, Neyret P. Medial retinaculum reefing for the treatment for patellar instability. Knee Surg Sports Traumatol Arthrosc. 2014;22(10):2505–12. 25059335 10.1007/s00167-014-3171-6

[36] Hautamaa PV, Fithian DC, Kaufman KR, Daniel DM, Pohlmeyer AM. Medial soft tissue restraints in lateral patellar instability and repair. Clin Orthop Relat Res. 1998;349:174–82. 10.1097/00003086-199804000-00021 9584380

[37] Nha KW, Kim HS, Cho ST, Bae JH, Jang KM, Kim SG. Arthroscopy-controlled medial reefing and lateral release for recurrent patellar dislocation: clinical, radiologic outcomes and complications. BMC Musculoskelet Disord. 2021;22(1):430. 33971864 10.1186/s12891-021-04300-x PMC8111772

[38] Panni AS, Vasso M, Cerciello S. Acute patellar dislocation. What to do? In., vol. 21: Springer; 2013: 275–278. 10.1007/s00167-012-2347-1 23242381

[39] He S. Advances in anatomy, biomechanics, injury and repair and reconstruction of the medial patellofemoral ligament. J Military Surgeon Southwest China. 2012;14(06):864–7.

[40] Miao XL, Zhang L, Zhuang HM, Zhao ZQ. Application of synthetic Substitute Materials on Reconstruction of Anterior Cruciate Ligament of Knee Joint in Exercise-induced Injury. In: *Advanced Materials Research: 2014*: Trans Tech Publ; 2014: 30–33.

[41] Khemka A, Lord SJ, Doyle Z, Bosley B, Al Muderis M. Minimally invasive medial patellofemoral ligament reconstruction for patellar instability using an artificial ligament: a two year follow-up. Knee. 2016;23(2):261–6. 26275579 10.1016/j.knee.2015.07.002

[42] Feng X, Wu T, Zou Y, Yan Q, Yang K, Xie T. Arthroscopic reconstruction of the medial patellofemoral ligament with double bundle of autologous thin femoral tendon combined with internal tibial tuberosity migration for recurrent patellar dislocation. Chin J Bone Joint Injury. 2019;34(01):97–9.

[43] Mehl J, Otto A, Comer B, Kia C, Liska F, Obopilwe E, Beitzel K, Imhoff AB, Fulkerson JP, Imhoff FB. Repair of the medial patellofemoral ligament with suture tape augmentation leads to similar primary contact pressures and joint kinematics like reconstruction with a tendon graft: a biomechanical comparison. Knee Surg Sports Traumatol Arthrosc. 2020;28(2):478–88. 31410528 10.1007/s00167-019-05668-z

